# Effect of Hand Hygiene Intervention in Community Kindergartens: A Quasi-Experimental Study

**DOI:** 10.3390/ijerph192214639

**Published:** 2022-11-08

**Authors:** Shiyang Wu, Richard Szewei Wang, Yu-Ni Huang, Thomas T. H. Wan, Tao-Hsin Tung, Bing-Long Wang

**Affiliations:** 1 Department of Public Health, Macau University of Science Technology, Macau 999078, China; 2 Affiliation Program of Data Analytics and Business Computing, Stern School of Business, New York University, New York, NY 10012, USA; 3 College of Public Health, National Taiwan University, Taipei 100, Taiwan; 4 School of Global Health Management and Informatics, University of Central Florida, Orlando, FL 32816, USA; 5 Evidence-Based Medicine Center, Taizhou Hospital of Zhejiang Province Affiliated to Wenzhou Medical University, Taizhou 317000, China; 6 School of Health Policy and Management, Chinese Academy of Medical Sciences & Peking Union Medical College, Beijing 100730, China

**Keywords:** hand hygiene, kindergarten, intervention effect, infectious disease control

## Abstract

This study aimed to evaluate the effect of hand hygiene interventions on the overall hand hygiene (HH) status of teaching instruction of hand hygiene in kindergartens, given the vulnerability of kindergarten children and their high risk due to infectious diseases and the current COVID-19 epidemic. We investigated the HH status of teachers from two kindergartens in the same community. The participants were recruited from 28 classes in both kindergartens. After completing the baseline survey, the intervention program consisted of three components: lectures on infectious diseases, lectures on HH, and seven-step hand washing techniques conducted in two kindergartens. The intervention program effectively increased teachers’ perceived disease susceptibility (*p* < 0.05), reduced the total bacterial colonization of children’s hands (*p* < 0.001), and improved the HH environment (*p* < 0.01). We recommend that health authorities or kindergartens adopt this HH intervention program to effectively improve the HH status in kindergartens and allow for preventive responses to the COVID-19 epidemic or other emerging infectious diseases.

## 1. Introduction

Since the COVID-19 outbreak at the end of 2019, it has remained severe for more than two years, with 594 million infections and 6.45 million deaths worldwide as of 23 August 2022 [1]. More than 14.4 million children in the United States were infected with COVID-19, indicating a continuous increase from 2% (April 2020) to 18.4% (March 2022) of the total cases. The cumulative number of deaths was 962, which accounted for 0.11% of the 900,000 deaths [2]. Children are a vulnerable population and require protection. Children have limited self-care abilities and often cannot wear masks effectively. Other infectious diseases, including diarrhea and pneumonia, are leading causes of pediatric deaths [3]. Globally, 1.25 million children aged <5 years died from diarrhea and acute lower respiratory infections in 2017 alone, which accounted for 22.7% of all deaths in this age group [4]. In China, children in this age group mainly receive early education in kindergartens—densely populated public places with numerous susceptible individuals. An outbreak can occur quickly in these spaces upon the emergence of an infectious disease [5,6,7].

Hand hygiene (HH) can effectively control the infection source, cut off the transmission route, and reduce the occurrence of infectious diseases [8]. Hand washing is among the most effective interventions for controlling the spread of microorganisms and preventing infections; moreover, it is easy and inexpensive to perform [9]. Therefore, the World Health Organization launched the healthcare campaign “Seconds Save Lives—Clean Your Hands” in 2021, which advocated for continuous improvement of HH in schools through healthcare and environmental cleaning to prevent the spread of pathogens; moreover, it recommended that good HH practices remain after the current COVID-19 pandemic [10]. Therefore, it is essential to implement HH interventions in kindergartens to reduce the incidence of infectious disease.

There has been a recent increase in research on effective HH interventions for kindergartens, with a study showing that promoting handwashing in daycare centers could reduce diarrhea cases by approximately 30% in high-income countries [11]. Moreover, HH interventions have shown reasonable control of hospital-acquired infections [12,13,14]. Additionally, HH educational courses in kindergartens can effectively reduce infection-related absences [15,16]. However, these studies also reported a high incidence of hand contamination among kindergarten children. As contaminated hands are one of the primary sources of potentially pathogenic bacteria causing respiratory and gastrointestinal infections in children, proper hand washing effectively reduces the spread of pathogenic bacteria from our hands and greatly reduces the spread of infection. Examining hands for bacteria can reflect whether children are washing their hands properly [17]. Therefore, the evaluation of hand hygiene interventions is also crucial. However, the effectiveness of HH interventions for kindergarten environmental sanitation and bacterial surveillance remains unclear. Therefore, the present study examined the effects of HH intervention on the overall HH status of kindergartens. Specifically, the study implemented an HH intervention and explored the HH status of teachers, bacterial status in children’s hands, and HH situation of kindergartens. Hence, our study facilitates the development of more cost-effective and efficient multimodal HH intervention strategies for improving HH and reducing the occurrence of infectious diseases in kindergartens.

## 2. Materials and Methods

### 2.1. Setting and Study Population

This quasi-experimental study was conducted with kindergarten teachers and children in Shenzhen between October 2019 and January 2020. The research objects were two kindergartens in a large community (population of approximately 100,000) in Shenzhen, China, with their teachers, children, and HH environment. The two kindergartens had nine primary classes (average age: 3 years), nine junior classes (average age: 4 years), and ten senior classes (average age: 5 years). Each classroom had handwashing facilities with sinks and faucets for children and teachers.

**Figure 1:**
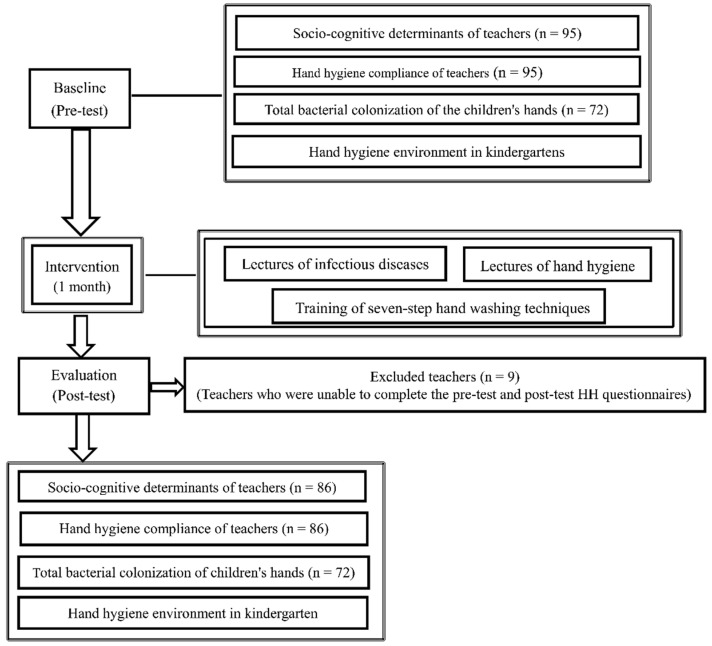
Flow diagram for the study design.

A total of 95teachers from 28 classes in two kindergartens were included in the intervention program for the pre-test (Figure 1). Given the limited experimental materials and the convenience of testing, we adopted stratified proportional sampling method and include 72 children as samples from a total of 913 children in 28 classes and conducted pre-test and post-test for hand bacteria colonization according to the “Chinese Technical Program for Disinfection Quality Testing of Medical Institutions and Kindergartens in Guangdong” [18].

### 2.2. Intervention Program

The intervention program was conducted over one month and consisted of three components: lecture on infectious diseases, lecture on hand hygiene, and the hand washing training in two kindergartens. This intervention program included a handwashing guideline based on an HH training session developed in Netherlands [19]. All intervention materials were pre-tested to be appropriate for reading or playing with Chinese children from mainland China [20].

Lectures on infectious diseases: Each teacher attended a 1.5 h infectious disease lecture after completing the baseline survey. A public health physician lectured on common childhood infectious diseases such as hand, foot, and mouth disease, chicken pox, mumps, and influenza, and covered prevention, treatment, and how to handle sick children.

Lectures on hand hygiene: After completing the baseline survey, each teacher in each kindergarten attended a 1 h lecture; a public health physician gave a lecture on the importance of handwashing in preventing infectious diseases, knowing why and when to wash hands, and how to do it properly.

Hand washing training: After the teachers had completed attending the infectious disease lecture and HH lecture, two children were selected from their respective classes to represent the class in a hands-on hand hygiene activity and learn the proper steps for hand washing. Finally, the teachers were required to conduct a hand hygiene session in their respective classes and teach children the proper steps for handwashing through audio stories, videos, and hands-on hand hygiene activity and learn the seven-step hand washing techniques. The training materials for the teacher lectures on HH knowledge were based on the training curriculum of the “Clean Hands, Happy Life” Shenzhen Kindergarten Hand Hygiene Promotion Project [21]. The teachers recorded the children’s learning and hand washing practice through photos for feedback to the research team.

### 2.3. Outcome Measurement

Before the HH intervention, all kindergarten teachers completed the “Kindergarten Teachers’ HH Questionnaire” on-site, with responses being collected on the same day. The questionnaire was based on a hand hygiene questionnaire, which was commonly used in hand hygiene surveys of caregivers in Netherlands daycare centers [19,20]. The questionnaire collected information regarding social demographics, socio-cognitive determinants, and HH compliance. Social-demographic questions included teachers’ demographic characteristics, including gender and age. Socio-cognitive determinants were designed based on the health belief model and divided into the following four dimensions: perceived disease susceptibility, perceived disease severity (the scale was 0–10, less possible to high possible), self-efficacy, and cue to action (the Likert scale was used, ranging from 1 (strongly disagree) to 7 (strongly agree)). HH compliance was assessed using 14 questions (the scale ranged from 0 (never wash hands) to 10 (always wash hands)).

We applied repeated measurements of teachers’ socio-cognitive determinants, HH compliance, children’s hand bacteria colonization, and HH environment. We examined bacterial colonization in the hands based on the guidelines of the “Technical Standard of Hospital Disinfection” [22]. We collected hand samples from the 72 children (one hand rubbing area was set at 24 cm^2^). Children’s hand bacteria were sampled before and after the intervention. The total number of bacterial colonies per square centimeter of the hands ≤8 cfu is qualified. Here, total bacterial colonization of 0–21 CFU/cm^2^, 21–42 CFU/cm^2^, 42–63 CFU/cm^2^, and ≥63 CFU/cm^2^ were defined as level “1”, “2”, “3”, “4”, respectively. The HH environment was specified as the number of hand sanitizers, available faucets, and paper towel devices in each class, which was recorded by the researcher using the “Kindergarten HH Environment Check List”. The checklist was designed based on the “Clean Hands, Happy Life” questionnaire of the Shenzhen Kindergarten Hand Hygiene Promotion Project [20,22]. Internal consistency was investigated using Cronbach’s α for each dimension of socio-cognitive determinants and overall HH compliance (Table 1). The Cronbach’s α is from 0.741 (cue to action) to 0.964 (perceived disease severity).

### 2.4. Statistical Analysis

Epidata 3.1 was used for coding and data entry. Moreover, double-entry proofreading was performed to ensure data accuracy. Statistical analyses were performed using SPSS version 24 For Windows. Data were described using mean, standard deviation (SD), interquartile deviation (IQR), median, frequency, and ratio. The chi-square test and t-test were used to determine differences in the teachers’ sociodemographic characteristics before and after the intervention. Nonparametric Mann–Whitney U and Kruskal–Wallis H tests were used to compare differences in socio-cognitive determinants, HH compliance of teachers, and total bacterial colonization of the children’s hand samples before and after the intervention. We used a nonparametric Wilcoxon signed-rank test to compare differences in the kindergarten HH environment before and after the intervention.

## 3. Results

In total, 95 (97.9%) and 86 (92.5%) teachers completed the pre-test and post-test HH questionnaires, respectively. Nine teachers who were unable to complete the post-test HH questionnaires were excluded. There were no significant differences in the pre- and post-intervention period for social demographics and teachers’ experiences of hand skin problems (dry hands and eczema) (Table 2).

### 3.1. Effect of the Intervention on the Teachers’ Socio-Cognitive Determinants and HH Compliance

Among the four dimensions of the teachers’ socio-cognitive determinants, perceived disease susceptibility, a deficient level at baseline (mean = 2.42), increased by 1.58 (65.2%) after the intervention. Contrastingly, the IQR increased by 3 (60%), which indicated a significant post-intervention increase in the teachers’ sensitivity to the fact that not washing their hands could endanger children’s health or promote infectious diseases (*p* < 0.05). However, the remaining dimensions of perceived disease severity, self-efficacy, and cue to action were at high levels before and after the intervention, with no significant differences being observed. Teachers perceived infectious disease as a serious matter and believed that there were more strategies or cues within their personal and external environments that drive them to wash their hands properly. The teachers’ overall HH compliance showed a post-intervention increase from 9.46 to 9.63, with a significant increase in HH compliance being specially observed “after coughing in the hands and/or sneezing” and “after blowing your nose”. Moreover, the teachers’ perceptions of each HH moment were generally consistent (IQR < 0.43). There was no significant difference in the teachers’ HH compliance before and after the intervention (*p* > 0.05) (Table 3).

### 3.2. Effect of the Intervention on Total Bacterial Colonization of the Children’s Hands

We collected samples from the 72 children’s hands for the bacterial colonization test. There was a significant post-intervention decrease in the overall total bacterial colonization of the children’s hands (median reduction from 3 to 1, *p* < 0.001), which indicated an overall two-level (67%) reduction in total bacterial colonization. The total bacterial colonization levels of the children’s hands in the primary, junior, and senior classes significantly decreased by three, three, and two levels, respectively (*p* < 0.05). The junior class showed the most significant increase in the passing rate of total bacterial colonization of hand samples. This indicated a significant post-intervention decrease in bacterial colonization counts of the hands of children in junior class, which showed the highest pre-intervention values. Taken together, the overall passing rate for total bacterial colonization of children’s hand samples increased from 15.28% to 37.5%, which indicated a more than double increase post-intervention in the number of participants’ proficiency in HH (Table 4).

### 3.3. Effect of the Intervention on the HH Environment

There was a significant post-intervention improvement in the kindergarten HH environment (*p* < 0.01). This demonstrated a post-intervention increase in the total consumption of hand sanitizer, the number of paper towel equipment, and available faucets. In addition, there was a slight and significant increase in the number of faucets available for children (IQR = 1) and teachers (IQR = 5), respectively. Additionally, there was a post-intervention increase in the mean of towels provided individually for each teacher, which indicated that some teachers began preparing their towels in the classroom (*p* < 0.05) (Table 5).

## 4. Discussion

Our findings demonstrated an increase in teachers’ perceived disease susceptibility, a significant decrease in the total bacterial colonization of the children’s hands, and a significant increase in the number of hand sanitizers, faucets, and paper towel devices for children and teachers in the HH environment, respectively. Health education is a simple and low-cost intervention that effectively increases the awareness of the importance of HH and improves the correctness and compliance with HH [23]. Compared with other studies on HH interventions for non-health workers [24,25], we first informed teachers regarding common childhood infectious diseases before the HH lecture. The teachers were made aware of the sources and transmission of infectious diseases and reminded to pay attention to the health status of children. The intervention program has improved their knowledge, attitudes, and beliefs about the relationship between infectious diseases and HH from the perspective of the health belief model. Additionally, after the lecture, the teachers become more willing to accept the HH education, learn how to wash their hands correctly, and improve HH compliance. Sensory learning is a vital teaching intervention strategy for early childhood education [26]. In this HH intervention for children, an illustrated audio story was used to convey the need for handwashing, which created a meaningful and effective learning environment. This facilitated the children’s understanding of the story to enhance their knowledge regarding proper handwashing [27].

Additionally, the children watched a video on handwashing exercises, learned and sang the seven-step handwashing song, and practiced handwashing under the guidance of teachers and public health specialists. This interactive and active learning approach deepens the children’s understanding of HH and promotes preventive behavior change [28]. Multiple factors contribute to improving infection control practices; multimodal intervention strategies significantly promote behavior change. As a critical step in a multimodal intervention strategy, health education was critically involved in HH behavior change as an intervention [8,29,30]. Qualitative and quantitative research on kindergarten HH health education intervention should be deepened in future research to provide a basis for developing multimodal intervention strategies.

Among the teachers’ socio-cognitive determinants, there was a significant post-intervention increase in the perceived disease susceptibility. This suggests that the infectious diseases and HH lectures effectively raised the teachers’ awareness. The teachers believed that not washing their hands would increase the risk of children contracting infectious diseases. The other three dimensions showed high scores before and after the intervention. The average HH compliance of the teachers was >9 before and after the intervention, with a non-significant post-intervention. Consistent with our results, a study found that self-reported HH compliance was higher than observed compliance; moreover, there was an overestimation of the self-reported compliance [31].

We found lower numbers of total bacterial colonization on children’s hands after intervention, the children’s HH passing rate increased from 15.28% to 37.5% after the intervention. This post-intervention improvement in HH was especially evident among children in the junior class, who showed the highest total bacterial colonization count in the hands. Arikan et al. found that a handwashing education intervention resulted in a 50% reduction in hand bacteria in preschool children; it reduced coliform bacteria’s growth rate on unclean hands [32]. This is consistent with our findings and suggests that our HH intervention effectively improved HH in children. Kindergarten children have a high chance of having contaminated hands, the primary sources of potentially pathogenic bacteria that cause respiratory and gastrointestinal infections [33]. Therefore, regular monitoring of hand bacteria in children is essential for improving HH compliance and preventing the transmission of infectious diseases, strategies to keep hands clean have always been of utmost importance in health care [34].

Regarding the environmental factor, we found that sufficient facilities, such as the number of hand sanitizers, faucets, and paper towel devices used by children and teachers, can be increased markedly after the intervention. In addition, previous studies indicated that the HH environment and available facilities could influence HH behavior changes [33,34,35,36]; for example, increasing faucets and hand sanitizer dispensers significantly improved children’s HH compliance, and children’s behavior [37,38]. Therefore, we strongly recommend that the HH environment factor be included in HH intervention programs.

This study has several limitations. First, we applied before-and-after cross-sectional intervention analysis, which does not imply causality. Second, self-reported compliance might likely overestimate actual compliance due to social desirability bias [39]. We did not individually follow up with the respondents at baseline due to the cluster-randomized nature of the trial and the anonymized survey. Third, this study consisted of two waves of data. Because of the lack of a true panel sample (with the same respondents), we must pool the data of two waves together. Hence, the use of pair-t tests was not feasible. Furthermore, by using anonymous questionnaires, we were unable to perform the paired-T tests. Finally, the lack of a control or a comparison group was also a limitation of the study. However, the present approach was to formulate a pooled cross-sectional panel analysis of the two waves of data. Thus, this study adopted a before-and-after cross-sectional intervention program. Ideally, the study could employ a propensity score matching and analysis to control the potential contributors to the variability of the sample.

## 5. Conclusions

Our findings confirmed that the intervention strategy of HH education effectively increased teachers’ perceived disease susceptibility, reduced total bacterial colonization of children’s hands, and improved the HH environment. Therefore, health authorities or kindergartens should consider adopting the same health education and intervention strategy to effectively improve the HH environment and allow epidemic prevention in response to the COVID-19 epidemic or other emerging infectious diseases. Moreover, to further address the limitations of the study, we suggest that future studies may consider adopting an experimental research design and consider using digital electronic devices to improve the reliability and validity of the study.

## Figures and Tables

**Table 1:** Example questions and scale reliability for socio-cognitive determinants and overall hand hygiene compliance among kindergarten teachers.

Variables	Numbers of Questions	Example Questions	Scale	Cronbach’s *α*
**Socio-cognitive determinants**				
Perceived disease susceptibility	2	What is the chance that a child in your class contracts an infection because you did not wash your hands?	Less possible (0) to High Possible (10)	0.946
Perceived disease severity	2	How serious are the possible consequences for a child when he/she contracts an infection?	Less possible (0) to High Possible (10)	0.964
Self-efficacy	5	I think washing hands according to the guidelines is something I can do consciously.	Likert scale (1 to 7)	0.847
Cue to action	10	We have all the materials and equipment needed for handwashing in our kindergarten.	Likert scale (1 to 7)	0.741
**Overall HH compliance**	14	Before the preparation of the lunch	Never wash hands (0) to Always wash hands (10)	0.828

**Table 2:** Comparison of teachers’ social characteristics before and after the intervention.

Variables	Before (*n* = 95)	After (*n* = 86)	*x^2^*/t	*p*-Value
	*n* (%)/Mean ± SD	*n* (%)/Mean ± SD		
**Class Category**			0.016	0.992
Primary-class	36 (37.9)	33 (38.4)		
Junior-Class	25 (26.3)	23 (26.7)		
Senior-class	34 (35.8)	30 (34.9)		
**Sex**				
Women	95 (100)	86 (100)		
Men	0 (0)	0 (0)		
**Age**	26.99 ± 6.45	27.7 ± 6.5	−0.735	0.463
**Number of years working as a teacher**	3.81 ± 2.93	4.04 ± 2.89	−0.533	0.595
**Education**			0.960	0.811
Junior high or lower	9 (5)	6 (7)		
High school or technical secondary school	30 (31.6)	29 (33.7)		
Junior college	49 (51.6)	42 (48.8)		
Bachelor or higher	7 (7.4)	9 (10.5)		
**Number of children under 14 years old living together**			0.103	0.950
None	55 (57.9)	48 (55.8)		
Only one	28 (29.5)	26 (30.2)		
Two or more	12 (12.7)	12 (14)		
**Suffer from dry hands**			0.111	0.739
Never	18 (18.9)	18 (20.9)		
Sometimes/always	77 (81.1)	68 (79.1)		
**Suffer from eczema**			0.509	0.476
Never	83 (87.4)	78 (90.7)		
Sometimes/always	12 (12.6)	8 (9.3)		

SD: Standard Deviation.

**Table 3:** Comparison of teachers’ social-cognitive determinants and hand hygiene compliance before and after the intervention.

Variables	Before (*n* = 95)		After (*n* = 86)		*p*-Value
	Mean	IQR	Mean	IQR	
**Socio-cognitive determinants**					
Perceived disease susceptibility	2.42	5.00	4.00	8.00	0.020 *
Perceived disease severity	8.89	1.00	8.70	1.50	0.911
Self-efficacy	6.76	0.20	6.81	0	0.132
Cue to action	6.45	0.80	6.37	0.90	0.966
**HH compliance**					
Before the preparation of the lunch	9.4	0	9.52	0	0.536
Before peeling of fruit	9.34	0	9.55	0	0.931
After coughing in the hands and/or sneezing	9.11	1	9.47	0	0.169
After blowing your nose	9.11	1	9.43	1	0.313
After changing a diaper with feces	9.48	0	9.72	0	0.969
After contacting with body fluids (saliva, vomit, blood, wound, urine, snot)	9.82	0	9.81	0	0.657
After playing outside	9.24	0	9.42	0.25	0.932
After contacting soiled textiles (dirty washcloths, towels)	9.51	0	9.55	0	0.851
After going to the toilet	9.72	0	9.77	0	0.899
Before preparing the bottle	9.41	0	9.68	0	0.257
Before you go eat yourself	9.61	0	9.74	0	0.396
Before you help a child with food	9.54	0	9.64	0	0.548
After wiping the nose of a child	9.48	0	9.71	0	0.570
After wiping a child’s butt	9.74	0	9.79	0	0.907
**Overall**	9.46	0.43	9.63	0.29	0.475

* *p* < 0.05; IQR: Interquartile range; IQR = Q3 − Q1.

**Table 4:** Comparison of total bacterial colonization levels of children’s hand before and after the intervention.

The Level of Total Bacterial Colonization	Before (*n* = 72)				After (*n* = 72)				*p*-Value
	Mean	Median	IQR	Passing Rate (%)	Mean	Median	IQR	Passing Rate (%)	
**Class category**									
Primary class (*n* = 17)	2.76	4	3	17.65	1.59	1	1	35.29	0.026 *
Junior class (*n* = 23)	2.83	4	3	8.70	1.96	1	3	34.78	0.044 *
Senior class (*n* = 32)	2.72	3	3	18.75	1.84	1	1.75	40.63	0.009 **
**Overall** (*n* = 72)	2.76	3	3	15.28	1.82	1	1	37.50	0.000 ***

The total bacterial colonization level: 1 = 0–21 cfu/cm^2^, 2 = 21–42 cfu/cm^2^, 3 = 42–63 cfu/cm^2^, 4 = 63 cfu/cm^2^ and above; * *p* < 0.05, ** *p* < 0.01, *** *p* < 0.001; IQR: Interquartile range; IQR = Q3 − Q1.

**Table 5:** Comparison of hand hygiene environment in kindergarten before and after the intervention.

Variables	Before		After		*p*-Value
	Mean	IQR	Mean	IQR	
**The number of sanitizers for children**	1.18	2.75	2.75	1.00	0.000 ***
The number of sanitizers for teachers	0.96	1.75	2.29	2.00	0.005 **
The number of faucets for children	2.86	5.00	5.43	1.00	0.000 ***
The number of faucets for teachers	2.25	1.00	4.50	5.00	0.004 **
The number of paper towel devices for children	0.71	1.00	1.11	0	0.008 **
The number of paper towel devices for teachers	0.18	0	0.64	1.00	0.002 **
The number of towels provided individually for each child	1.21	0	1.18	0	0.564
The number of towels provided individually for each teacher	0	0	0.18	0	0.025 *
The number of towels used by teachers for classroom cleaning	4.82	1.00	4.71	3.00	0.66
The number of towels shared by children and teachers	0	0	0	0	1.00

* *p* < 0.05, ** *p* < 0.01, *** *p* < 0.001; IQR: Interquartile range; IQR = Q3 − Q1.

## Data Availability

The datasets used and analyzed during the current study are not publicly available but are available from the corresponding author on reasonable request.
